# Patterns of utilization and effects of hospital-specific factors on physical, occupational, and speech therapy for critically ill patients with acute respiratory failure in the USA: results of a 5-year sample

**DOI:** 10.1186/s13054-019-2467-9

**Published:** 2019-05-16

**Authors:** Clare C. Prohaska, Peter D. Sottile, Amy Nordon-Craft, Matt D. Gallagher, Ellen L. Burnham, Brendan J. Clark, Michael Ho, Tyree H. Kiser, R. William Vandivier, Wenhui Liu, Margaret Schenkman, Marc Moss

**Affiliations:** 1 Department of Medicine, University of Colorado School of Medicine, Aurora, CO 80045 USA; 2 Division of Pulmonary Sciences and Critical Care Medicine, University of Colorado School of Medicine, Box C272, 12700 E 19th Ave, Aurora, CO 80045 USA; 3 Department of Physical Therapy, University of Colorado Hospital, Aurora, CO 80045 USA; 4 University of Colorado Hospital, Aurora, CO 80045 USA; 5 Division of Cardiology, University of Colorado School of Medicine, Aurora, CO 80045 USA; 6 Department of Clinical Pharmacy, University of Colorado School of Pharmacy and Pharmaceutical Sciences, Aurora, CO 80045 USA; 7 VA Eastern Colorado Health Care System, Aurora, CO 80045 USA

**Keywords:** Early rehabilitation, Intensive care outcomes, Acute respiratory failure

## Abstract

**Background:**

Timely initiation of physical, occupational, and speech therapy in critically ill patients is crucial to reduce morbidity and improve outcomes. Over a 5-year time interval, we sought to determine the utilization of these rehabilitation therapies in the USA.

**Methods:**

We performed a retrospective cohort study utilizing a large, national administrative database including ICU patients from 591 hospitals. Patients over 18 years of age with acute respiratory failure requiring invasive mechanical ventilation within the first 2 days of hospitalization and for a duration of at least 48 h were included.

**Results:**

A total of 264,137 patients received invasive mechanical ventilation for a median of 4.0 [2.0–8.0] days. Overall, patients spent a median of 5.0 [3.0–10.0] days in the ICU and 10.0 [7.0–16.0] days in the hospital. During their hospitalization, 66.5%, 41.0%, and 33.2% (95% CI = 66.3–66.7%, 40.8–41.2%, 33.0–33.4%, respectively) received physical, occupational, and speech therapy. While on mechanical ventilation, 36.2%, 29.7%, and 29.9% (95% CI = 36.0–36.4%, 29.5–29.9%, 29.7–30.1%) received physical, occupational, and speech therapy. In patients receiving therapy, their first physical therapy session occurred on hospital day 5 [3.0–8.0] and hospital day 6 [4.0–10.0] for occupational and speech therapy. Of all patients, 28.6% (95% CI = 28.4–28.8%) did not receive physical, occupational, or speech therapy during their hospitalization. In a multivariate analysis, patients cared for in the Midwest and at teaching hospitals were more likely to receive physical, occupational, and speech therapy (all *P* < 0.05). Of patients with identical covariates receiving therapy, there was a median of 61%, 187%, and 70% greater odds of receiving physical, occupational, and speech therapy, respectively, at one randomly selected hospital compared with another (median odds ratio 1.61, 2.87, 1.70, respectively).

**Conclusions:**

Physical, occupational, and speech therapy are not routinely delivered to critically ill patients, particularly while on mechanical ventilation in the USA. The utilization of these therapies varies according to insurance coverage, geography, and hospital teaching status, and at a hospital level.

**Electronic supplementary material:**

The online version of this article (10.1186/s13054-019-2467-9) contains supplementary material, which is available to authorized users.

## Background

Intensive care unit-acquired weakness (ICU-AW) occurs commonly in patients with acute respiratory failure requiring mechanical ventilation [1]. ICU-AW is associated with multiple deleterious outcomes including increased ICU and hospital mortality, prolonged ICU and hospital stay, prolonged duration of mechanical ventilation, and pharyngeal dysfunction that may increase the susceptibility to develop aspiration pneumonia [2–5]. After hospital discharge, ICU survivors are also limited in their physical function, and these decrements in activities of daily living may persist 5 years after hospital discharge [6–10]. As a result, critical care professionals have focused on preventing and treating ICU-AW with the early initiation of physical, occupational, and speech therapy [11–17].

Despite increased awareness about the potential benefits of physical rehabilitation, the frequency, timing, and duration of physical, occupational, and speech therapy for patients with acute respiratory failure requiring mechanical ventilation are relatively unknown. Prior national surveys in the USA and abroad have reported the opinions of healthcare professionals about their perceived practice, yet did not examine actual practice patterns [18–20]. Several point prevalence studies have been published; however, they involved a small number of hospitals and only collected data for a few days [21–26]. Finally, no prior studies examined the effects of insurance status, regional variation, or teaching status of the hospital on physical, occupational, and speech therapy utilization.

Therefore, we sought to determine the national utilization of physical, occupational, and speech therapy for patients with acute respiratory failure requiring mechanical ventilation.

## Methods

### Study design

We performed a retrospective cohort study of the Premier Incorporated Perspective Database, a cohort of over 7 million unique intensive care unit (ICU) admissions nationally from January 2010 through December 2014. The database has been described in detail previously [27–30]. The Colorado Multiple Institutional Review Board approved this study.

### Patient population

Adult patients 18 years or older who were admitted to the ICU with acute respiratory failure were included. Acute respiratory failure was defined as patients who received invasive mechanical ventilation within the first 2 days of hospitalization, and received at least two consecutive days of mechanical ventilation. Invasive mechanical ventilation was defined by the presence of 39 different possible charges for intubation or mechanical ventilation on each day (see Additional file 1: Table S1). Patients who were transferred from an outside hospital, had repeated admissions in the dataset, or had a hospital stay of less than 4 days were excluded. Physical, occupational, and speech therapy utilization was determined by the presence of specific hospital charge codes (see Additional file 2: Table S2) on each day. Patient characteristics included age, gender, race, and insurance coverage. Categories of insurance coverage were Medicare, Medicaid, private insurance, self-pay, other, or charity. Hospital characteristics included geographic region, hospital size, rural versus urban setting, and academic versus private teaching status. Geographic regions were defined as Northeast, South, Midwest, and West (Additional file 3: Figure S1).

### Analysis

Our primary outcome measures were utilization, time to initiation, and total number of sessions of physical, occupational, and speech therapy during the hospitalization and while on mechanical ventilation.

We fit a Bayesian model with Markov chain Monte Carlo (MCMC) method to the outcomes [31]. To account for hospital-level variation, as well as hospital-level correlation between the three outcomes, a joint mixed effect model with logistic link function was used to evaluate the association between patient insurance coverage (Medicare, Medicaid, private insurance, self-pay, charity, or other with Medicaid as the reference group), geographic region (four regions with Midwest as the reference group), and hospital teaching status (yes/no with teaching as the reference group) upon the utilization of physical, occupational, and speech therapy. Three outcomes were jointed with correlated hospital random effect. Patient demographic factors such as age, gender, race, and location (urban versus rural) were adjusted in the model. Thirty thousand-iteration simulation with 5000 burning iterations in MCMC procedure was used. We also calculated hospital median odds ratios for each outcome.

The median odds ratio (MOR) measures variation in prescribing patterns between hospitals, with a value always ≥ 1. It is the median of all theoretically derived odds ratios between two patients with identical covariate values drawn randomly from any two hospitals; in our case, for the likelihood of therapy prescription at a hospital with higher rates of therapy ordered to a patient at a hospital with a lower level of therapy prescription. In effect, this compares the effect of hospital heterogeneity on therapy prescription to that of the patient-level factors as it uses the same scale as odds ratios obtained for patient-level covariates. A MOR of 1 signifies that there is no difference in therapy prescription between two patients with the same covariates at different hospitals. A higher MOR indicates a higher probability of therapy prescription at one hospital versus another [32].

To evaluate the role of individual physicians in ordering physical, occupational, and speech therapy, we determined the variance in prescribing patterns at the hospital level, assuming that physicians who practice together likely have similar practice patterns. We controlled for this variance as a random effect in our models by using a median odds ratio.

The Python (3.6) scientific ecosystem, which includes the Pandas (23.0), SciPy (1.1), and Statsmodels (0.9) open source software libraries, as well as SAS 9.4, was used for all data and statistical analysis [33–37].

## Results

**Fig. 1:**
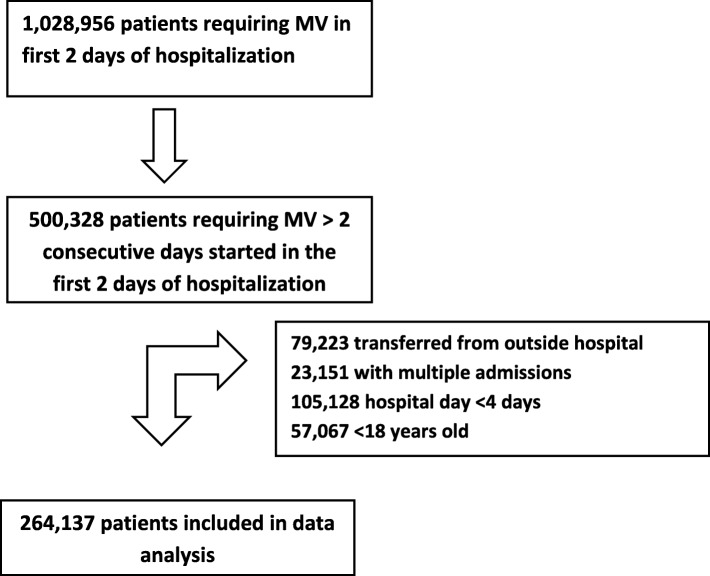
Inclusion flow diagram. Exclusion criteria were not mutually exclusive categories

Over 1.40 million patients required mechanical ventilation during the first 2 days of hospitalization. Of these, 500,328 received at least two consecutive days of mechanical ventilation. Of these patients, 79,223 were transferred from an outside hospital, 23,151 had multiple admissions in the Premier database, 105,128 had a hospital stay less than 4 days, and 51,067 were less than 18 years old. Therefore, a total of 264,137 unique patient admissions from 591 hospitals were included in the final analysis (Fig. 1).

Study population demographics can be found in Table 1. Medicare was the most common insurance coverage at 56.3%; 19.9% had private insurance, 12.9% had Medicaid, 5.9% were self-pay, 4.1% had other insurance, and < 1.0% received charity care. A total of 46.4% of patients were located in the South, 21.0% located in the Midwest, 17.9% located in the West, and 14.7% located in the Northeast. Of the patients included in our study, the median length of intubation was 4.0 days [2.0–8.0], ICU length of stay 5.0 days [3.0–10.0], and hospital length of stay 10.0 days [7.0–16.0] (Table 1). Of the 591 hospitals included in the study, the median number of hospital beds was 234.0 [122.5, 384.0].

**Table 1:** Demographic information

Characteristic	Received PT (*N* = 175,611)	Did not receive PT (*N* = 88,526)	Received OT (*N* = 108,312)	Did not receive OT (*N* = 155,825)	Received ST (*N* = 87,770)	Did not receive ST (*N* = 176,367)	All subjects (*N* = 264,137)
Mean age (SD)	63.0 (16.3)	61.0 (17.2)	62.0 (16.8)	62.5 (16.6)	62.6 (17.2)	62.2 (16.4)	62.3 (16.6)
% Male	54.3	55.3	55.1	54.3	55.7	54.1	54.6
% White	68.8	64.9	67.9	67.3	67.1	67.7	67.5
Insurance (%)							
Medicare	100,852 (57.4%)	47,948 (54.2%)	59,535 (55.0%)	89,265 (57.3%)	49,448 (56.3%)	99,352 (56.3%)	56.3%
Private	35,138 (20.0%)	17,517 (19.8%)	22,682 (20.9%)	29,973 (19.2%)	16,494 (18.8%)	36,161 (20.5%)	19.9%
Medicaid	21,583 (12.3%)	12,425 (14.0%)	14,059 (13.0%)	19,949 (12.8%)	12,117 (13.8%)	21,891 (12.4%)	12.8%
Self-pay	9361 (5.3%)	6262 (7.1%)	6291 (5.8%)	9332 (6.0%)	5071(5.8%)	10,552(6.0%)	5.9%
Other	7230 (4.1%)	3542 (4.0%)	4965 (4.6%)	5807 (3.7%)	3852 (4.4%)	6920 (3.9%)	4.1%
Charity	1447 (0.8%)	832 (0.9%)	780 (0.7%)	1499 (1.0%)	788 (0.9%)	1491 (0.8%)	0.9%
Median # of beds	417	390	423	391	423	399	234
% Urban	90.8	89.0	91.9	89.0	91.5	89.6	77.0
Region (%)							
Midwest	31,648 (18.0%)	14,384 (16.2%)	25,259 (23.3%)	20,773 (13.3%)	15,695 (17.9%)	30,337 (17.2%)	21.0%
Northeast	29,744 (16.9%)	13,828 (15.6%)	15,027 (13.9%)	28,545 (18.3%)	14,550 (16.6%)	29,022 (16.5%)	14.7%
South	85,611 (48.8%)	45,608 (51.5%)	49,306 (45.5%)	81,913 (52.6%)	41,050 (46.8%)	90,169 (51.1%)	46.4%
West	28,608 (16.3%)	14,706 (16.6%)	18,720 (17.3%)	24,594 (15.8%)	16,475 (18.8%)	26,839 (15.2%)	17.9%
% Academic	49.0	43.9	52.1	43.9	50.3	45.8	29.9
Length of MV (median) [25–75% quartile]	4.0 [2.0–8.0]	4.0 [3.0–7.0]	4.0 [2.0–8.0]	3.0 [2.0–6.0]	5.0 [3.0–9.0]	3.0 [2.0–6.0]	4.0 [2.0–8.0]
ICU LOS (median) [25–75% quartile]	6.0 [3.0–10.0]	5.0 [3.0–9.0]	6.0 [4.0–12.0]	5.0 [3.0–8.0]	7.0 [4.0–13.0]	5.0 [3.0–8.0]	5.0 [3.0–10.0]
Hospital LOS (median) [25–75% quartile]	12.0 [8.0–18.0]	9.0 [7.0–13.0]	13.0 [9.0–20.0]	10.0 [7.0–15.0]	14.0 [9.0–22.0]	10.0 [7.0–14.0]	10.0 [7.0–16.0]

### Primary analysis

Overall, 175,611 patients (66.5%, 95% CI = 66.3–66.7%) received physical therapy, 108,312 patients (41.0%, 95% CI = 40.8–41.2%) received occupational therapy, and 87,770 patients (33.2%, 95% CI = 33.0–33.4%) received speech therapy during their hospitalization. Of patients who received rehabilitation during their hospitalization, 36.2% (95% CI = 36.0–36.4%) of patients received physical therapy, 29.7% (95% CI = 29.5–29.9%) of patients received occupational therapy, and 29.9% (95% CI = 29.7–31.1%) of patients received speech therapy and mechanical ventilation on the same day. Table 2 further details when therapy was initiated and how frequently therapy was delivered while patients were still hospitalized.

**Table 2:** Therapy utilization

Outcomes	Physical therapy	Occupational therapy	Speech therapy
Received therapy during hospitalization	175,611 (66.5%)	108,312 (41.0%)	87,770 (33.2%)
Received therapy and MV on the same day	63,639 (36.2%)	32,183 (29.7%)	26,270 (29.9%)
Time to initiation of therapy (days after intubation) [25–75% quartile]	4.0 [2.0–7.0]	5.0 [3.0–9.0]	5.0 [3.0–9.0]
Length of therapy (days) [25–75% quartile]	3.0 [2.0–6.0]	2.0 [1.0–4.0]	3.0 [1.0–4.0]
Median odds ratio (SD)	1.61 (0.03)	2.87 (0.11)	1.70 (0.03)

**Fig. 2:**
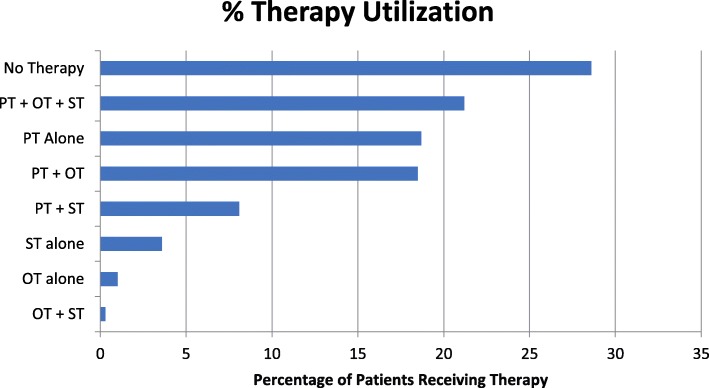
Therapy ordering patterns. Overall, 75,527 patients (28.6%) did not receive any therapy. The most common ordering patterns were the combination of physical, occupational, and speech therapy [55,914 patients (21.2%)] and physical therapy alone [49,297 patients (18.7%)], followed by physical and occupational therapy [48,910 patients (18.5%)]. PT physical therapy, OT occupational therapy, ST speech therapy

Overall, 28.6% (95% CI = 28.4–28.8%) of patients did not receive any type of rehabilitation. The most frequent therapy combination was physical, occupational, and speech therapy, ordered in 21.2% (95% CI = 21.0–21.4%) of patients. Physical therapy alone was ordered in 18.6% (95% CI = 18.4–18.8%), and physical and occupational therapy were ordered together in 18.5% (95% CI = 18.3–18.7%) of patients (Fig. 2).

### Multivariable analysis

The results of a multivariable regression model controlling for payor type, hospital geographic location, and hospital teaching status are reported in Table 3. For physical, occupational, and speech therapy, patients were more likely to receive therapy in the Midwest and at teaching hospitals [OR 1.20 (95% CI 1.18–1.22%), 1.81 (95% CI 1.47–2.23%), 1.16 (95% CI 1.07–1.26%), respectively]. The median odds ratio was 1.61 for physical therapy, 2.87 for occupational therapy, and 1.70 for speech therapy. This suggests that there was significantly greater variation between hospitals with regard to therapy prescription that cannot be measured by hospital-level characteristics alone. There were 42,049 physicians who admitted the 264,137 patients in our study. Individual physicians were responsible for a median admission of 2 patients [interquartile range 1.0–6.0, max 1704].

**Table 3:** Multivariate analysis: association of insurance status, geographic region, and teaching status on therapy orders

Variable		Physical therapy		Occupational therapy		Speech therapy	
		*N*	OR (95% CI)	*N*	OR (95% CI)	*N*	OR (95% CI)
Payor	Medicaid	21,583	–	14,059	–	12,117	–
	Medicare	100,852	1.02 (0.99–1.05)	59,535	0.96* (0.94–0.97)	49,448	0.87* (0.86–0.89)
	Private	35,138	1.04* (1.01–1.07)	22,682	1.01 (0.98–1.03)	16,494	0.80* (0.79–0.81)
	Self-pay	9361	0.88* (0.86–0.89)	6291	0.89* (0.85–0.93)	5071	0.88* (0.86–0.89)
	Other	7230	1.13* (1.12–1.15)	4965	1.20* (1.14–1.25)	3852	0.98 (0.93–1.03)
	Charity	1447	0.94 (0.86–1.03)	780	0.90* (0.84–0.95)	788	0.88*(0.80–0.97)
Region	Midwest	31,648	–	25,259	–	15,695	–
	West	29,744	0.85* (0.74–0.98)	15,027	0.57* (0.43–0.77)	14,550	1.09 (0.93–1.28)
	Northeast	85,611	0.76* (0.66–0.88)	49,306	0.26* (0.19–0.36)	41,050	0.79* (0.67–0.94)
	South	28,608	0.79* (0.70–0.88)	18,720	0.32* (0.25–0.40)	16,475	0.79* (0.70–0.90)
Teaching status	Private	89,597	–	51,832	–	43,661	–
	Academic	86,014	1.20* (1.8–1.22)	56,480	1.81* (1.47–2.23)	44,109	1.16* (1.07–1.26)

Reference groups included Medicaid insurance status, Midwest region, and private teaching status. *Indicates results at a 0.05 significance level

## Discussion

Critically ill patients with respiratory failure who require invasive mechanical ventilation receive physical, occupational, and speech therapy in a variable manner. Patients were unlikely to receive rehabilitation while on mechanical ventilation. Typically, therapy is initiated several days after hospital admission, and patients receive only a few days of therapy during their hospitalization. Our study found that despite increasing awareness of early mobilization, physical rehabilitation does not happen as early in a hospital stay as may be possible. Furthermore, we found that there are variations with how rehabilitation is delivered related to insurance status, geographic region, and hospital teaching status.

With the increasing awareness of ICU-AW, there has been increased interest in the early utilization of these therapies. Previous studies were either surveys that measured perceptions of delivery of care, only examined limited numbers of hospitals, or were point prevalence studies a few days throughout the year. These previous study designs might not accurately reflect utilization trends in the USA. For example, two previous studies sent surveys to ICU providers, who estimated that between 39 and 47% patients on mechanical ventilation underwent early mobilization [18, 19]. However, point prevalence studies suggest that this is a significant overestimation. In a recent study by Jolley et al. of 42 ICUs over 2 days, only 16% of mechanically ventilated patients achieved out of bed mobility [22]. Other international surveys from countries such as France, Germany, and the UK estimate early mobility at 40%, 59%, and 52%, respectively [20], while point prevalence studies from Germany, Australia, and New Zealand reported that 0–3% of patients receive physical therapy while on mechanical ventilation [23–26]. Our data showed that 24.1% of patients received physical therapy and mechanical ventilation codes on the same day, an intermediate value between these two estimates. It is possible that with increased awareness of ICU-AW, providers are ordering these therapies more frequently while patients still require mechanical ventilation, or very soon after extubation.

Bakhru et al. surveyed ICUs in four different countries. There was wide variation amongst dedicated physical therapists (34% in the USA versus 92% in the UK), as well as a nurse to patient ratios (typically 1:1 in the UK and up to 1:4 in France). They found that early mobilization was significantly associated with a lower nurse to patient staffing ratio, presence of dedicated physical therapists (therapist who is primarily assigned to patients in the ICU), and multidisciplinary rounds (rounds consisting of physicians, nurses, and other healthcare workers such as social workers, physical or respiratory therapists, or pharmacists). Early mobility was not associated with academic affiliation [20].

Despite a robust investigation of early physical therapy, less understanding of the role of occupational and speech therapy exists. One single-center study regarding the independent use of occupational therapy demonstrated a utilization rate of 30% in ICU patients [38]. We found that during an entire hospital stay, this rate was only 41.0% and reduced to only 12.2% of patients on mechanical ventilation. Similarly, we found that speech therapy was ordered in 33.2% of all patients. If not recognized early, patients may be vulnerable to post-extubation dysphagia, which has been shown to lead to poor patient outcomes and longer hospital stays in certain patient populations [39, 40]. In an observational study, the prevalence of dysphagia was 84% in patients recovering from critical illness; however, only 25% of these patients received a speech therapy consultation [41]. Speech therapy may be an underutilized resource, and further studies regarding long-term follow-up of early interventions are warranted.

To our knowledge, no previous studies have shown differences in the utilization of physical, occupational, or speech therapy based on insurance status, geographic variation, or hospital teaching status. There was significant variation between insurance coverage and therapy implementation; if the utilization of therapy leads to improved outcomes and is cost-effective, this may lead to broader therapy utilization in the future. Since the conduct of this study, the USA enacted the Affordable Care Act. Whether this policy change had any effect on ordering patterns of physical rehabilitation is unknown, and this could be explored in future studies. Given the observational nature of our study, it was impossible to factor in clinician decision making. It is therefore unknown if patient insurance status had any effect on whether the clinician decided to order rehabilitation services.

Geographic differences may reflect local or regional hospital culture related to different training of providers, highlighted by median odds ratios calculated for each therapy type. Although there has been no published research reporting how geography plays a role in physical rehabilitation in the ICU, there have been previous studies that show patients who must travel longer distances have decreased rates of healthcare utilization [42, 43]. It could be extrapolated that patients in the Midwest are generally located in more rural settings, and thus, providers in this region are more cognizant of limited exposure to healthcare and thus order physical rehabilitation more frequently than other regions. Studies detailing early mobility in the USA are typically performed at major cities or academic centers [12–17], so it is unclear how their results are generalizable to more rural settings or other countries. Furthermore, we found that teaching hospitals were more likely than non-teaching hospitals to provide therapy, possibly indicating that during training, providers are increasingly utilizing physical, occupational, and speech therapy. Unfortunately, there are no studies currently available that report other countries’ physical rehabilitation patterns with respect to geography or insurance coverage. It remains unclear if countries with nationalized healthcare have any difference in physical rehabilitation ordering patterns [23–26].

There are several important limitations to this observational study. First, our data was collected between 2010 and 2014. It is possible that the utilization of these therapies has changed in recent years since awareness of early mobility has increased. Second, we used therapy codes as a marker for therapy intervention. Some patients received therapy codes that might have only been evaluated by a therapist and not actually received any therapy. Therefore, our findings may overestimate the true delivery of rehabilitative interventions. Third, it is possible that early consultation is not always warranted, for example, speech therapy consultation while a patient remains on mechanical ventilation. Currently, there are no ways to identify patients who would receive additional benefit from early physical, occupational, and speech therapy, and further research is indicated. Fourth, given the nature of this data set, it is unclear if certain clinicians order physical rehabilitation more frequently than others. It is possible that certain providers are more consistent about ordering physical rehabilitation early into a hospital stay compared to other physicians, which may also vary with geography or hospital teaching status. There are likely other hospital factors that influence physical rehabilitation ordering patterns, such as patient to provider ratio or number of patient comorbidities. Fifth, the data utilized in this study was generated only from hospitals in the USA, and results in other countries, particularly those with nationalized healthcare systems, may be different. Finally, this is an observational study and thus causal associations cannot be determined. Additional studies are necessary to see how prescribing patterns are affecting long-term patient outcomes, how increased multidisciplinary care can be achieved in the ICU, and how these patterns are changing over time.

## Conclusions

Critically ill patients receive physical, occupational, and speech therapy in a variable manner during their hospitalization, and only a minority while on mechanical ventilation. On average, rehabilitation is initiated several days after admission, and only few sessions are completed prior to discharge. Patients with private insurance, located in certain areas of the country or cared for at teaching hospitals are most likely to receive early rehabilitation. Further studies are needed to determine how these differences affect long-term patient morbidity and recovery.

## Additional files

Additional file 1: **Table S1.** Mechanical ventilation charge codes. (DOCX 15 kb)

Additional file 2: **Table S2.** Physical, occupational, and speech therapy utilization codes. (DOCX 26 kb)

Additional file 3: **Figure S1.** Geographic regions. (DOCX 65 kb)

## Abbreviations

ICU: Intensive care unit
ICU-AW: Intensive care unit-associated weakness
MCMC method: Markov chan Monte Carlo method
MOR: Median odds ratio
OT: Occupational therapy
PT: Physical therapy
ST: Speech therapy
