# Supplementary material for: Patterns of utilization and effects of hospital-specific factors on physical, occupational, and speech therapy for critically ill patients with acute respiratory failure in the USA: results of a 5-year sample
Source: Crit Care. 2019 May 16;23:175. doi: 10.1186/s13054-019-2467-9 (PMC6524324; doi:10.1186/s13054-019-2467-9)
Supplement: Supplementary file 1 — Table S1. Mechanical ventilation charge codes. (DOCX 15 kb) [file 13054_2019_2467_MOESM1_ESM.docx]

| 270270086600000 | VENTILATOR HI FREQUENCY SETUP |
| --- | --- |
| 270270088900000 | VENTILATOR SETUP |
| 270270089300000 | VENTILATOR PRESSURE SETUP |
| 290290093620000 | VENTILATOR DAILY |
| 410410940040000 | VENTILATOR NURSING FACILITY PER DAY |
| 410410940050000 | VENTILATOR MANAGEMENT CARE PLAN IN HOME |
| 410412000580000 | VENTILATION MANUAL PER 15 MIN |
| 410412000590000 | VENTILATOR ASSESSMENT |
| 410412000600000 | VENTILATOR MAINTENANCE |
| 410412000610000 | VENTILATOR STANDBY |
| 410412000620000 | VENTILATOR TRANSPORT |
| 410412000630000 | VENTILATOR VOLUME MONITOR |
| 410412000640000 | VENTILATOR WEANING PARAMETERS |
| 410412946560000 | VENTILATOR PER SHIFT (8HRS) |
| 410412946560001 | VENTILATOR PER SHIFT (8HRS) |
| 410412946570000 | VENTILATOR PER SHIFT (8HRS) |
| 410412946570002 | VENTILATOR PER SHIFT (8HRS) |
| 410412946570003 | VENTILATOR VOLUME PER HR |
| 410412946570004 | VENTILATOR PER SHIFT (8HRS) |
| 410412946570005 | VENTILATOR PER SHIFT (8HRS) |
| 410412946570007 | VENTILATOR PER SHIFT (8HRS) |
| 410412946570009 | VENTILATOR PER SHIFT (8HRS) |
| 970976946560000 | PF VENTILATOR FIRST DAY |
| 970976946570000 | PF VENTILATOR SUBSEQUENT DAY |
| 999999041682009 | PATIENT CARE ICU W/MECHANICAL VENT 24 HRS OR LESS |
| 270270027280000 | HOLDER ENDOTRACHEAL |
| 270270027290000 | HOLDER ENDOTRACHEAL |
| 270270027600000 | HUMIDIFIER |
| 270270053610000 | TRAY RESPIRATORY |
| 270270055490000 | TUBE ENDOTRACHEAL |
| 270270055570000 | TUBE ENDOTRACHEAL |
| 270270055580000 | TUBE ENDOTRACHEAL |
| 270270990340000 | TUBE ENDOTRACHEAL |
| 360360315000000 | EMERGENCY INTUBATION |
| 360450315000000 | EMERGENCY INTUBATION |
| 360490315000000 | EMERGENCY INTUBATION |
| 360750315000000 | EMERGENCY INTUBATION |
| 410410000150000 | EXTUBATION ENDOTRACHEAL |
| 970975315000000 | PF EMERGENCY INTUBATION |
