# Supplementary material for: Patterns of utilization and effects of hospital-specific factors on physical, occupational, and speech therapy for critically ill patients with acute respiratory failure in the USA: results of a 5-year sample
Source: Crit Care. 2019 May 16;23:175. doi: 10.1186/s13054-019-2467-9 (PMC6524324; doi:10.1186/s13054-019-2467-9)
Supplement: Supplementary file 2 — Table S2. Physical, occupational, and speech therapy utilization codes. (DOCX 26 kb) [file 13054_2019_2467_MOESM2_ESM.docx]

**PT Codes**

| std_chg_code | std_chg_desc |
| --- | --- |
| 420420000010000 | PT PER DAY |
| 420420000020000 | PT STAT FEE |
| 420420000030000 | PT STERILE TECHNIQUE |
| 420420000050000 | PT ORTHO TECH CALLBACK FEE |
| 420420000060000 | PT ORTHO TECH STAT FEE |
| 420420000070000 | PT FOLLOWUP |
| 420420000080000 | PT ORTHO TECH 15 MIN |
| 420420000090000 | PT AIDE/ADDL PERSON |
| 420420000100000 | PT HOME VISIT |
| 420420000110000 | PT IN ROOM VISIT |
| 420420000120000 | PT EDUCATION PATIENT 15 MIN |
| 420420000130000 | PT HOT PACK/E-STIM/ULTRASOUND 15 MIN |
| 420420000140000 | PT EQUIPMENT CHECK 15 MIN |
| 420420000150000 | PT HOT PACK/E-STIM 15 MIN |
| 420420000160000 | PT HOT PACK/TRACTION/ULTRASOUND 15 MIN |
| 420420000170000 | PT EDUCATION FAMILY 15 MIN |
| 420420000180000 | PT EDUCATION BACK CARE 1 HR |
| 420420000190000 | PT CONFERENCE 10 MIN |
| 420420000210000 | PT CONFERENCE 30 MIN |
| 420420000220000 | PT CONFERENCE 20 MIN |
| 420420000230000 | PT CONFERENCE 45 MIN |
| 420420000240000 | PT CONFERENCE DISCHARGE 15 MIN |
| 420420000250000 | PT EDUCATION PATIENT 30 MIN |
| 420420000260000 | PT ULTRASOUND W/E-STIM 15 MIN |
| 420420000270000 | PT EDUCATION BACK CARE 15 MIN |
| 420420000300000 | PT CONFERENCE 15 MIN |
| 420420000330000 | PT CONFERENCE 1 HR |
| 420420001832008 | PT ULTRASOUND,TOP APPL,WOUND CARE,PER DAY |
| 420420290000000 | PT APPLICATION CAST HALO BODY |
| 420420290100000 | PT APPLICATION RISSJACKET BODY |
| 420420290150000 | PT APPLICATION RISSJACKET LOCAL BODY W/HEAD |
| 420420290200000 | PT APPLICATION TURNBUCKLE JACKET BODY ONLY |
| 420420290250000 | PT APPLICATION TURNBUCKLE JACKET INCLUDE HEAD |
| 420420290350000 | PT APPLICATION BODY CAST SHOULDER/HIPS |
| 420420290400000 | PT APPLICATION BODY CAST SHOULDER/HIPS/HEAD |
| 420420290440000 | PT APPLICATION BODY CAST SHOULDER/HIPS/THIGH |
| 420420290460000 | PT APPLICAT BODY CAST SHOULDER TO HIPS BOTH THIGHS |
| 420420290490000 | PT APPLICATION FIGURE EIGHT PLASTER CAST |
| 420420290550000 | PT APPLICATION CAST SPICA SHOULDER |
| 420420290580000 | PT APPLICATION CAST PLASTER VELPEAU |
| 420420290650000 | PT APPLICATION LONG ARM CAST |
| 420420290750000 | PT APPLICATION FOREARM CAST |
| 420420290850000 | PT APPLICATION CAST HAND/FOREARM |
| 420420290860000 | PT APPLICATION CAST FINGER |
| 420420291050000 | PT APPLICATION LONG ARM SPLINT |
| 420420291250000 | PT APPLICATION FOREARM SPLINT |
| 420420291260000 | PT APPLICATION SPLINT SHORT ARM DYNAMIC |
| 420420291300000 | PT APPLICATION FINGER SPLINT STATIC |
| 420420291310000 | PT APPLICATION FINGER SPLINT DYNAMIC |
| 420420292000000 | PT STRAPPING THORAX |
| 420420292200000 | PT STRAPPING LOW BACK |
| 420420292400000 | PT STRAPPING SHOULDER |
| 420420292600000 | PT STRAPPING ELBOW OR WRIST |
| 420420292800000 | PT STRAPPING HAND OR FINGER |
| 420420293050000 | PT APPLICATION CAST SPICA HIP/LEG |
| 420420293250000 | PT APPLICATION CAST SPICA HIP/LEGS |
| 420420293450000 | PT APPLICATION LONG LEG CAST |
| 420420293550000 | PT APPLICATION LONG LEG CAST WALKING/AMBULATORY |
| 420420293580000 | PT APPLICATION LONG LEG CAST BRACE |
| 420420293650000 | PT APPLICATION CAST CYLINDER |
| 420420294050000 | PT APPLICATION SHORT LEG CAST |
| 420420294250000 | PT APPLICATION SHORT LEG CAST WALKING/AMBULATORY |
| 420420294350000 | PT APPLICATION PATELLAR TENDON BEARING (PTB) CAST |
| 420420294400000 | PT ADD WALKER TO CAST |
| 420420294450000 | PT APPLICATION CAST RIGID CONTACT LEG |
| 420420294500000 | PT APPLICATION CAST CLUBFOOT |
| 420420295050000 | PT APPLICATION LONG LEG SPLINT |
| 420420295150000 | PT APPLICATION LOWER LEG SPLINT |
| 420420295200000 | PT STRAPPING HIP |
| 420420295300000 | PT STRAPPING KNEE |
| 420420295400000 | PT STRAPPING ANKLE AND/OR FOOT |
| 420420295500000 | PT STRAPPING TOES |
| 420420295800000 | PT STRAPPING UNNA BOOT |
| 420420295810000 | PT APPLY VENOUS WOUND COMPRESSION BELOW KNEE |
| 420420295900000 | PT STRAPPING DENIS BROWNE |
| 420420297990000 | PT STRAPPING/CASTING UNLIST PROC |
| 420420909010000 | PT BIOFEEDBACK 15 MIN |
| 420420909110000 | PT BIOFEEDBACK W/EMG 15 MIN |
| 420420970100000 | PT HOT/COLD PACK 15 MIN |
| 420420970100001 | PT COLD PACK 15 MIN |
| 420420970100002 | PT CRYOTHERAPY |
| 420420970100003 | PT HOT PACK 15 MIN |
| 420420970100004 | PT HOT/COLD PACK |
| 420420970120000 | PT TRACTION MECHANICAL |
| 420420970120001 | PT TRACTION CERVICAL |
| 420420970120002 | PT TRACTION PELVIC |
| 420420970120003 | PT TRACTION LUMBAR |
| 420420970120004 | PT TRACTION INTERMITTENT |
| 420420970120005 | PT TRACTION CHECK MAJOR |
| 420420970120006 | PT TRACTION CHECK MINOR |
| 420420970120007 | PT SUSPENSION BALANCED |
| 420420970140000 | PT ELECTRICAL STIM UNATTENDED 15 MIN |
| 420420970140001 | PT TENS 15 MIN |
| 420420970140002 | PT TENS FOLLOW UP/CHECK 15 MIN |
| 420420970160000 | PT VASOPNEUMATIC TREATMENT 15 MIN |
| 420420970160001 | PT VASOPNEUMATIC FITTING 15 MIN |
| 420420970160002 | PT VASOPNEUMATIC FITTING 30 MIN |
| 420420970180000 | PT PARAFFIN BATH 15 MIN |
| 420420970200000 | PT MICROWAVE THERAPY |
| 420420970220000 | PT WHIRLPOOL |
| 420420970220001 | PT WHIRLPOOL BODY STERILE W/DRESSING |
| 420420970220002 | PT WHIRLPOOL EXTREMITY 15 MIN |
| 420420970220003 | PT WHIRLPOOL BODY 15 MIN |
| 420420970220005 | PT WHIRLPOOL BODY STERILE 15 MIN |
| 420420970220006 | PT HYDROTHERAPY 15 MIN |
| 420420970220007 | PT WHIRLPOOL EXTREMITY STERILE W/DRESSING |
| 420420970220008 | PT WHIRLPOOL EXTREMITY STERILE 15 MIN |
| 420420970240000 | PT DIATHERMY THERAPY(MICROWAVE) 15 MIN |
| 420420970240001 | PT DIATHERMY THERAPY(MICROWAVE) |
| 420420970260000 | PT INFRARED THERAPY |
| 420420970280000 | PT ULTRAVIOLET THERAPY |
| 420420970320000 | PT ELECTRICAL STIM ATTENDED 15 MIN |
| 420420970320001 | PT HIGH VOLTAGE GALVANIC STIM 15 MIN |
| 420420970330000 | PT IONTOPHORESIS 15 MIN |
| 420420970340000 | PT CONTRAST BATH 15 MIN |
| 420420970350000 | PT ULTRASOUND 15 MIN |
| 420420970360000 | PT HUBBARD TANK 15 MIN |
| 420420970360001 | PT HUBBARD TANK STERILE 15 MIN |
| 420420970390000 | PT PER MODALITY 15 MIN |
| 420420970390001 | PT PER MODALITY 30 MIN |
| 420420970390002 | PT MEDCOSONOLATOR 15 MIN |
| 420420970390003 | PT PHONOPHORESIS 15 MIN |
| 420420970390004 | PT PER MODALITY 45 MIN |
| 420420970390005 | PT PER MODALITY 25 MIN |
| 420420970390006 | PT PER MODALITY 1 HR |
| 420420970390020 | PT FLUIDOTHERAPY 15 MIN |
| 420420971100000 | PT EXERCISE THERAPEUTIC 15 MIN |
| 420420971100001 | PT EXERCISE BACK 15 MIN |
| 420420971100002 | PT EXERCISE THERAPEUTIC 30 MIN |
| 420420971100003 | PT EXERCISE THERAPEUTIC 20 MIN |
| 420420971100004 | PT BTE/WORK SIMULATOR 15 MIN |
| 420420971100005 | PT EXERCISE ISOKINETIC 30 MIN |
| 420420971100006 | PT EXERCISE KINETIC 30 MIN |
| 420420971100007 | PT EXERCISE ISOKINETIC 15 MIN |
| 420420971100008 | PT EXERCISE RANGE OF MOTION 15 MIN |
| 420420971100009 | PT EXERCISE KINETIC 15 MIN |
| 420420971100010 | PT EXERCISE CARDIAC 15 MIN |
| 420420971120000 | PT NEUROMUSCULAR RE-ED 15 MIN |
| 420420971120001 | PT MOBILITY TRAINING 15 MIN |
| 420420971120002 | PT NEUROMUSCULAR RE-ED PED 15 MIN |
| 420420971130000 | PT AQUATIC THERAPY/EXERCISE 15 MIN |
| 420420971130001 | PT AQUATIC THERAPY/EXERCISE 30 MIN |
| 420420971160000 | PT GAIT TRAINING 15 MIN |
| 420420971160001 | PT AMBULATION 15 MIN |
| 420420971160002 | PT GAIT TRAINING ADDL PERSON 15 MIN |
| 420420971160003 | PT GAIT TRAINING 20 MIN |
| 420420971160004 | PT GAIT TRAINING 30 MIN |
| 420420971220000 | PT TRACTION MANUAL 15 MIN |
| 420420971240000 | PT MASSAGE 15 MIN |
| 420420971240001 | PT MASSAGE ICE 15 MIN |
| 420420971390000 | PT PROCEDURE UNLISTED 15 MIN |
| 420420971390001 | PT BANDAGING 15 MIN |
| 420420971390002 | PT CPM ADJUSTMENT 15 MIN |
| 420420971390003 | PT CPM APPLICATION 15 MIN |
| 420420971390004 | PT CPM CHECK 15 MIN |
| 420420971390005 | PT DEBR & DRESSING 15 MIN |
| 420420971390006 | PT DEBR 15 MIN |
| 420420971390007 | PT DRESSING CHANGE LG 30 MIN |
| 420420971390008 | PT DRESSING CHANGE SM 15 MIN |
| 420420971390009 | PT DRESSING STERILE 15 MIN |
| 420420971390010 | PT SPLINT CONSTRUCTION 1 HR |
| 420420971390011 | PT SPLINT CONSTRUCTION 15 MIN |
| 420420971390012 | PT SPLINT CONSTRUCTION 30 MIN |
| 420420971390013 | PT TAPING 15 MIN |
| 420420971390014 | PT TILT TABLE 15 MIN |
| 420420971390015 | PT WOUND CARE 15 MIN |
| 420420971400000 | PT MANUAL THERAPY 15 MIN |
| 420420972500000 | PT MYOFASCIAL/SOFT TISSUE MOBILIZATION |
| 420420972600000 | PT MANIPULATION REGIONAL |
| 420420972610000 | PT MANIPULATION ADDL AREA |
| 420420972650000 | PT JOINT MOBILIZATION 15 MIN |
| 420420975040000 | PT ORTHOTIC FITTING/TRAINING 15 MIN |
| 420420975040001 | PT ORTHOTIC FITTING 15 MIN |
| 420420975040002 | PT ORTHOTIC TRAINING 15 MIN |
| 420420975040003 | PT ORTHOTIC TRAINING 30 MIN |
| 420420975040004 | PT BRACE FITTING 15 MIN |
| 420420975200000 | PT PROSTHETIC TRAINING 15 MIN |
| 420420975200001 | PT PROSTHETIC TRAINING 30 MIN |
| 420420975300000 | PT THERAPEUTIC ACTIVITY 15 MIN |
| 420420975300001 | PT FUNCTIONAL ACTIVITY 15 MIN |
| 420420975330000 | PT SENSORY INTEGRATIVE TECHNIQUE 15 MIN |
| 420420975330001 | PT SENSORY PERCEPTION 15 MIN |
| 420420975350000 | PT HOME INSTRUCTION/PROGRAM 15 MIN |
| 420420975350001 | PT ADL 15 MIN |
| 420420975350002 | PT ADL 30 MIN |
| 420420975370000 | PT COMMUNITY/WORK REINTEGRATION 15 MIN |
| 420420975370001 | PT WORK CAPACITY ANALYSIS 15 MIN |
| 420420975420000 | PT WHEELCHAIR TRAINING 15 MIN |
| 420420975420001 | PT TRANSFER TRAINING 15 MIN |
| 420420975420002 | PT TRANSFER TRAINING 30 MIN |
| 420420975450000 | PT WORK HARDENING 1ST 2 HRS |
| 420420975450001 | PT WORK HARDENING 1 HR |
| 420420975450002 | PT WORK HARDENING 15 MIN |
| 420420975450003 | PT WORK HARDENING 30 MIN |
| 420420975460000 | PT WORK HARDENING EACH ADDL HR |
| 420420975980000 | PT DEBRIDEMENT SELECT PER SESSION EA ADDL 20CM |
| 420420976010000 | PT DEBRIDEMENT SELECT PER SESSION 1ST 20CM OR LESS |
| 420420976010001 | PT DEBRIDEMENT SELECTIVE PER SESSION |
| 420420976020000 | PT DEBRIDEMENT NON-SELECT W/O ANES PER SESSION |
| 420420976020001 | PT DEBRIDEMENT NON-SELECTIVE PER SESSION |
| 420420976050000 | PT NEGATIVE PRESSURE WOUND THERAPY DME 50CM OR < |
| 420420976060000 | PT NEGATIVE PRESSURE WOUND THERAPY DME 50CM OR > |
| 420420976070000 | PT NEGATIVE PRESSURE WOUND THER DISP EQ 50CM OR < |
| 420420976080000 | PT NEGATIVE PRESSURE WOUND THER DISP EQ 50CM OR > |
| 420420976100000 | PT LOW FREQ NON-CONT NON-THERMAL ULTRASOUND P/DAY |
| 420420977030000 | PT ORTHOTIC/PROSTHETIC CHECKOUT 15 MIN |
| 420420977500000 | PT PERFORMANCE TEST 15 MIN |
| 420420977500001 | PT FUNCTIONAL CAPACITY TEST PER HR |
| 420420977550000 | PT ASSISTIVE TECHNOLOGY ASSESSMENT 15 MIN |
| 420420977700000 | PT COGNITIVE TRAINING 15 MIN |
| 420420977800000 | PT ACUPUNCTURE W/O E-STIM |
| 420420977810000 | PT ACUPUNCTURE W/E-STIM |
| 420420977990000 | PT REHAB PROCEDURE UNLISTED |
| 420420977990001 | PT REHAB 15 MIN |
| 420420977990002 | PT PROCEDURE UNLISTED |
| 420420977990003 | PT MISC |
| 420423971500000 | PT/OT EXERCISE GROUP 15 MIN |
| 420423971500001 | PT/OT EXERCISE GROUP 30 MIN |
| 420423971500002 | PT/OT EXERCISE GROUP 1 HR |
| 420423971500003 | PT/OT EXERCISE GROUP POOL 15 MIN |
| 420424970010001 | PT EVAL 15 MIN |
| 420424970010002 | PT EVAL 20 MIN |
| 420424970010003 | PT EVAL 30 MIN |
| 420424970010004 | PT EVAL 45 MIN |
| 420424970010005 | PT EVAL HOME 30 MIN |
| 420424970010006 | PT EVAL ISOKINETIC 30 MIN |
| 420424970010007 | PT EVAL ORTHOTIC 15 MIN |
| 420424970010008 | PT EVAL PRE OP 15 MIN |
| 420424970010009 | PT EVAL SCREENING 15 MIN |
| 420424970010010 | PT EVAL TENS 15 MIN |
| 420424970010011 | PT EVAL WHEELCHAIR 15 MIN |
| 420424970010015 | PT EVAL 1 HR |
| 420424970010016 | PT CONSULT 15 MIN |
| 420424970010100 | PT EVALUATION |
| 420424970020000 | PT RE-EVAL 15 MIN |
| 420424970020001 | PT RE-EVAL 30 MIN |
| 420424970020100 | PT RE-EVALUATION |
| 430430294350000 | OT APPLICATION PATELLAR TENDON BEARING (PTB) CAST |
| 430430977700002 | OT SENSORY PERCEPTION 15 MIN |

**OT Codes:**

| 430430000050000 | OT STERILE TECHNIQUE |
| --- | --- |
| 430430000060000 | OT INDEPENDENT ACTIVITY |
| 430430000070000 | OT CHECK |
| 430430000080000 | OT FOLLOWUP |
| 430430000090000 | OT AIDE/ADDL PERSON |
| 430430000100000 | OT CONFERENCE 10 MIN |
| 430430000110000 | OT CONFERENCE 15 MIN |
| 430430000120000 | OT CONFERENCE 20 MIN |
| 430430000130000 | OT CONFERENCE 30 MIN |
| 430430000140000 | OT CONFERENCE ADDL 15 MIN |
| 430430000150000 | OT CONFERENCE 1 HR |
| 430430000160000 | OT CONFERENCE DISCHARGE 15 MIN |
| 430430000170000 | OT CONFERENCE FAMILY 15 MIN |
| 430430000180000 | OT EDUCATION 1 HR |
| 430430000190000 | OT HOME VISIT |
| 430430000200000 | OT IN ROOM VISIT |
| 430430000220000 | OT EDUCATION FAMILY 15 MIN |
| 430430000230000 | OT EDUCATION 15 MIN |
| 430430000240000 | OT EDUCATION 30 MIN |
| 430430001832008 | OT ULTRASOUND,TOP APPL,WOUND CARE,PER DAY |
| 430430290000000 | OT APPLICATION CAST HALO BODY |
| 430430290100000 | OT APPLICATION RISSJACKET BODY |
| 430430290150000 | OT APPLICATION RISSJACKET LOCAL BODY W/HEAD |
| 430430290200000 | OT APPLICATION TURNBUCKLE JACKET BODY ONLY |
| 430430290250000 | OT APPLICATION TURNBUCKLE JACKET INCLUDE HEAD |
| 430430290350000 | OT APPLICATION BODY CAST SHOULDER/HIPS |
| 430430290400000 | OT APPLICATION BODY CAST SHOULDER/HIPS/HEAD |
| 430430290440000 | OT APPLICATION BODY CAST SHOULDER/HIPS/THIGH |
| 430430290460000 | OT APPLICA BODY CAST SHOULDER TO HIPS BOTH THIGHS |
| 430430290490000 | OT APPLICATION FIGURE EIGHT PLASTER CAST |
| 430430290550000 | OT APPLICATION CAST SPICA SHOULDER |
| 430430290580000 | OT APPLICATION CAST PLASTER VELPEAU |
| 430430290650000 | OT APPLICATION LONG ARM CAST |
| 430430290750000 | OT APPLICATION FOREARM CAST |
| 430430290850000 | OT APPLICATION CAST HAND/FOREARM |
| 430430290860000 | OT APPLICATION CAST FINGER |
| 430430291050000 | OT APPLICATION LONG ARM SPLINT |
| 430430291250000 | OT APPLICATION FOREARM SPLINT |
| 430430291260000 | OT APPLICATION SPLINT SHORT ARM DYNAMIC |
| 430430291300000 | OT APPLICATION FINGER SPLINT STATIC |
| 430430291310000 | OT APPLICATION FINGER SPLINT DYNAMIC |
| 430430292000000 | OT STRAPPING THORAX |
| 430430292200000 | OT STRAPPING LOW BACK |
| 430430292400000 | OT STRAPPING SHOULDER |
| 430430292600000 | OT STRAPPING ELBOW OR WRIST |
| 430430292800000 | OT STRAPPING HAND OR FINGER |
| 430430293050000 | OT APPLICATION CAST SPICA HIP/LEG |
| 430430293250000 | OT APPLICATION CAST SPICA HIP/LEGS |
| 430430293450000 | OT APPLICATION LONG LEG CAST |
| 430430293550000 | OT APPLICATION LONG LEG CAST WALKING/AMBULATORY |
| 430430293580000 | OT APPLICATION LONG LEG CAST BRACE |
| 430430293650000 | OT APPLICATION CAST CYLINDER |
| 430430294050000 | OT APPLICATION SHORT LEG CAST |
| 430430294250000 | OT APPLICATION SHORT LEG CAST WALKING/AMBULATORY |
| 430430294400000 | OT ADD WALKER TO CAST |
| 430430294450000 | OT APPLICATION CAST RIGID CONTACT LEG |
| 430430294500000 | OT APPLICATION CAST CLUBFOOT |
| 430430295050000 | OT APPLICATION LONG LEG SPLINT |
| 430430295150000 | OT APPLICATION LOWER LEG SPLINT |
| 430430295200000 | OT STRAPPING HIP |
| 430430295300000 | OT STRAPPING KNEE |
| 430430295400000 | OT STRAPPING ANKLE AND/OR FOOT |
| 430430295500000 | OT STRAPPING TOES |
| 430430295800000 | OT STRAPPING UNNA BOOT |
| 430430295810000 | OT APPLY VENOUS WOUND COMPRESSION BELOW KNEE |
| 430430295900000 | OT STRAPPING DENIS BROWNE |
| 430430297990000 | OT STRAPPING/CASTING UNLIST PROC |
| 430430909010000 | OT BIOFEEDBACK 15 MIN |
| 430430970100000 | OT HOT/COLD PACK |
| 430430970100001 | OT HOT PACK 15 MIN |
| 430430970100002 | OT COLD PACK 15 MIN |
| 430430970120000 | OT TRACTION MECHANICAL |
| 430430970140000 | OT ELECTRICAL STIM UNATTENDED |
| 430430970160000 | OT VASOPNEUMATIC TREATMENT |
| 430430970160001 | OT VASOPNEUMATIC FITTING 15 MIN |
| 430430970160002 | OT VASOPNEUMATIC TREATMENT 15 MIN |
| 430430970180000 | OT PARAFFIN BATH THERAPY |
| 430430970180001 | OT PARAFFIN BATH 15 MIN |
| 430430970220000 | OT WHIRLPOOL THERAPY |
| 430430970220001 | OT WHIRLPOOL EXTREMITY 15 MIN |
| 430430970220003 | OT WHIRLPOOL BODY 15 MIN |
| 430430970220004 | OT HYDROTHERAPY |
| 430430970240000 | OT DIATHERMY THERAPY(MICROWAVE) 15 MIN |
| 430430970240001 | OT DIATHERMY THERAPY(MICROWAVE) |
| 430430970260000 | OT INFRARED THERAPY |
| 430430970280000 | OT ULTRAVIOLET THERAPY |
| 430430970320000 | OT ELECTRICAL STIM ATTENDED 15 MIN |
| 430430970320001 | OT TENS 15 MIN |
| 430430970330000 | OT IONTOPHORESIS 15 MIN |
| 430430970340000 | OT CONTRAST BATH 15 MIN |
| 430430970350000 | OT ULTRASOUND 15 MIN |
| 430430970360000 | OT HUBBARD TANK 15 MIN |
| 430430970360001 | OT HUBBARD TANK STERILE 15 MIN |
| 430430970390000 | OT PER MODALITY 15 MIN |
| 430430970390001 | OT PER MODALITY PED 15 MIN |
| 430430970390002 | OT FLUIDOTHERAPY 15 MIN |
| 430430971100000 | OT EXERCISE THERAPEUTIC 15 MIN |
| 430430971100001 | OT EXERCISE THERAPEUTIC 30 MIN |
| 430430971100002 | OT EXERCISE UPPER EXTREMITY 15 MIN |
| 430430971100003 | OT EXERCISE RANGE OF MOTION 15 MIN |
| 430430971100004 | OT EXERCISE KINETIC 30 MIN |
| 430430971100005 | OT EXERCISE KINETIC 15 MIN |
| 430430971100006 | OT EXERCISE ISOKINETIC 15 MIN |
| 430430971100007 | OT EXERCISE HAND 15 MIN |
| 430430971100008 | OT EXERCISE CARDIAC 15 MIN |
| 430430971100009 | OT EXERCISE AEROBIC 1 HR |
| 430430971120000 | OT NEUROMUSCULAR RE-ED 15 MIN |
| 430430971120001 | OT MOTOR COORDINATION 15 MIN |
| 430430971120002 | OT MOTOR COORDINATION 1 HR |
| 430430971130000 | OT AQUATIC THERAPY/EXERCISE 15 MIN |
| 430430971160000 | OT GAIT TRAINING 15 MIN |
| 430430971220000 | OT TRACTION MANUAL |
| 430430971240000 | OT MASSAGE 15 MIN |
| 430430971240001 | OT MASSAGE ICE 15 MIN |
| 430430971390001 | OT DRESSING SM 15 MIN |
| 430430971390002 | OT DRESSING MED 15 MIN |
| 430430971390003 | OT DRESSING LG 15 MIN |
| 430430971390004 | OT SPLINT CONSTRUCTION 15 MIN |
| 430430971390005 | OT SPLINT CONSTRUCTION 30 MIN |
| 430430971390006 | OT DEBR 15 MIN |
| 430430971390007 | OT TAPING 15 MIN |
| 430430971400000 | OT MANUAL THERAPY 15 MIN |
| 430430972500000 | OT MYOFASCIAL/SOFT TISSUE MOBILIZATION |
| 430430972600000 | OT MANIPULATION REGIONAL |
| 430430972610000 | OT MANIPULATION ADDL |
| 430430972650000 | OT JOINT MOBILIZATION 15 MIN |
| 430430975040000 | OT ORTHOTIC FITTING/TRAINING 15 MIN |
| 430430975040001 | OT ORTHOTIC ADJUSTMENT 15 MIN |
| 430430975040002 | OT ORTHOTIC TRAINING 15 MIN |
| 430430975040003 | OT ORTHOTIC TRAINING 30 MIN |
| 430430975040004 | OT ORTHOTIC FITTING 15 MIN |
| 430430975200000 | OT PROSTHETIC TRAINING 15 MIN |
| 430430975200001 | OT PROSTHETIC TRAINING 30 MIN |
| 430430975300000 | OT THERAPEUTIC ACTIVITY 15 MIN |
| 430430975300001 | OT FUNCTIONAL ACTIVITY 15 MIN |
| 430430975330000 | OT SENSORY INTEGRATIVE TECHNIQUE 15 MIN |
| 430430975350001 | OT ADL 15 MIN |
| 430430975350002 | OT ADL 30 MIN |
| 430430975350003 | OT ADL ADVANCED 15 MIN |
| 430430975350004 | OT HOME PROGRAM 15 MIN |
| 430430975370000 | OT WORK CAPACITY ANALYSIS |
| 430430975370001 | OT COMMUNITY REINTEGRATION OUTING 15 MIN |
| 430430975370002 | OT WORK SIMULATION 1 HR |
| 430430975370003 | OT WORK CAPACITY ANALYSIS 15 MIN |
| 430430975370004 | OT WORK CAPACITY ANALYSIS 1 HR |
| 430430975370005 | OT WORK CAPACITY ANALYSIS ADDL 30 MIN |
| 430430975370006 | OT WORK SIMULATION 30 MIN |
| 430430975370008 | OT WORK CAPACITY ANALYSIS 30 MIN |
| 430430975370009 | OT COMMUNITY/WORK REINTEGRATION 15 MIN |
| 430430975420000 | OT WHEELCHAIR TRAINING 15 MIN |
| 430430975450000 | OT WORK HARDENING 1ST 2 HRS |
| 430430975450001 | OT WORK HARDENING 30 MIN |
| 430430975450002 | OT WORK HARDENING 1 HR |
| 430430975460000 | OT WORK HARDENING ADDL HR |
| 430430975980000 | OT DEBRIDEMENT SELECT PER SESSION EA ADDL 20CM |
| 430430976010000 | OT DEBRIDEMENT SELECT PER SESSION 1ST 20CM OR LESS |
| 430430976010001 | OT DEBRIDEMENT SELECTIVE PER SESSION |
| 430430976020000 | OT DEBRIDEMENT NON-SELECT W/O ANES PER SESSION |
| 430430976020001 | OT DEBRIDEMENT NON-SELECTIVE PER SESSION |
| 430430976050000 | OT NEGATIVE PRESSURE WOUND THERAPY DME 50CM OR < |
| 430430976060000 | OT NEGATIVE PRESSURE WOUND THERAPY DME 50CM OR > |
| 430430976070000 | OT NEGATIVE PRESSURE WOUND THER DISP EQ 50CM OR < |
| 430430976080000 | OT NEGATIVE PRESSURE WOUND THER DISP EQ 50CM OR > |
| 430430976100000 | OT LOW FREQ NON-CONT NON-THERMAL ULTRASOUND P/DAY |
| 430430977030000 | OT ORTHOTIC/PROSTHETIC CHECKOUT 15 MIN |
| 430430977500001 | OT FUNCTIONAL CAPACITY TEST 15 MIN |
| 430430977500002 | OT BTE/WORK SIMULATOR TEST 15 MIN |
| 430430977500003 | OT SPECIAL REPORT 15 MIN |
| 430430977500004 | OT FUNCTIONAL CAPACITY TEST 30 MIN |
| 430430977500005 | OT FUNCTIONAL CAPACITY TEST 1 HR |
| 430430977500006 | OT FUNCTIONAL CAPACITY TEST 2 HRS 30 MIN |
| 430430977500007 | OT FUNCTIONAL CAPACITY TEST 6 HRS |
| 430430977500008 | OT FUNCTIONAL CAPACITY TEST ADDL HR |
| 430430977550000 | OT ASSISTIVE TECHNOLOGY ASSESSMENT 15 MIN |
| 430430977700000 | OT COGNITIVE TRAINING 15 MIN |
| 430430977700001 | OT COGNITIVE TRAINING 30 MIN |
| 430430977700003 | OT DEVELOPMENT 15 MIN |
| 430430977800000 | OT ACUPUNCTURE W/O E-STIM |
| 430430977810000 | OT ACUPUNCTURE W/E-STIM |
| 430430977990000 | OT REHAB PROCEDURE UNLISTED |
| 430430977990001 | OT REHAB 15 MIN |
| 430430977990002 | OT PROCEDURE UNLISTED |
| 430430977990003 | OT MISC |
| 430430977990004 | OT PROCEDURE UNLISTED 15 MIN |
| 430433000250000 | OT EDUCATION GROUP 30 MIN |
| 430433971500000 | OT THERAPY GROUP 15 MIN |
| 430433971500001 | OT MOTOR COORDINATION GROUP 15 MIN |
| 430433971500003 | OT THERAPY GROUP PED 15 MIN |
| 430433971500004 | OT WORK SIMULATION GROUP 1 HR |
| 430433971500005 | OT WORK SIMULATION GROUP ADDL 30 MIN |
| 430433971500006 | OT COGNITIVE TRAINING GROUP 15 MIN |
| 430433971500007 | OT NEUROMUSCULAR RE-ED GROUP 15 MIN |
| 430434970030000 | OT EVAL 15 MIN |
| 430434970030001 | OT EVAL 1 HR |
| 430434970030002 | OT EVAL 10 MIN |
| 430434970030003 | OT EVAL 20 MIN |
| 430434970030004 | OT EVAL 30 MIN |
| 430434970030005 | OT EVAL 45 MIN |
| 430434970030006 | OT EVAL ADDL 15 MIN |
| 430434970030007 | OT EVAL DISABILITY 15 MIN |
| 430434970030008 | OT EVAL HAND THERAPY 15 MIN |
| 430434970030009 | OT EVAL HOME 15 MIN |
| 430434970030010 | OT EVAL PED 15 MIN |
| 430434970030011 | OT EVAL SCREENING 15 MIN |
| 430434970030012 | OT EVAL SENSORY 15 MIN |
| 430434970030013 | OT EVAL WHEELCHAIR 15 MIN |
| 430434970030014 | OT EVAL HOME 1 HR |
| 430434970030015 | OT EVAL ISOKINETIC 15 MIN |
| 430434970030020 | OT CONSULT 15 MIN |
| 430434970030023 | OT EVALUATION |
| 430434970040000 | OT RE-EVAL 15 MIN |
| 430434970040004 | OT RE-EVAL 30 MIN |
| 430434970040100 | OT RE-EVALUATION |

**ST Codes:**

| std_chg_code | std_chg_desc |
| --- | --- |
| 440440000010000 | ST EDUCATION PATIENT/FAMILY 15 MIN |
| 440440000020000 | ST CONFERENCE 60 MIN |
| 440440000030000 | ST CONFERENCE 15 MIN |
| 440440000040000 | ST CONFERENCE 30 MIN |
| 440440000050000 | ST HOME VISIT |
| 440440000060000 | ST IN ROOM VISIT |
| 440440000070000 | ST HOME THERAPY PROGRAM |
| 440440925020000 | ST EXAM OTOLARYNGOLOGIC |
| 440440925040000 | ST BINOCULAR MICROSCOPY |
| 440440925070000 | ST TREATMENT SPEECH |
| 440440925070001 | ST TREATMENT SPEECH 15 MIN |
| 440440925070002 | ST TREATMENT VOICE 15 MIN |
| 440440925070003 | ST TREATMENT SPEECH PED 15 MIN |
| 440440925070004 | ST TREATMENT SPEECH PED 90 MIN |
| 440440925070005 | ST TREATMENT AUDITORY |
| 440440925070006 | ST TREATMENT LANGUAGE 15 MIN |
| 440440925070007 | ST TREATMENT SPEECH PED 60 MIN |
| 440440925070008 | ST TREATMENT COGNITIVE |
| 440440925070009 | ST TREATMENT COGNITIVE 15 MIN |
| 440440925100000 | ST TREATMENT AURAL W/IMPLANT |
| 440440925110000 | ST NASOPHARYNGOSCOPY |
| 440440925120000 | ST NASAL FUNCTION STUDY |
| 440440925160000 | ST FACIAL NERVE FUNCTION STUDY |
| 440440925160001 | ST ORAL/FACIAL MOTOR SEQUENCE 15 MIN |
| 440440925200000 | ST LARYNGEAL FUNCTION STUDY |
| 440440925210000 | EVALUATION OF SPEECH FLUENCY |
| 440440925220000 | EVALUATION OF SPEECH SOUND PRODUCTION |
| 440440925230000 | EVAL SPEECH SOUND PRODUCTION W/EVAL COMP & EXPRESS |
| 440440925240000 | BEHAVIOR & QUALITATIVE ANALYSIS VOICE & RESONANCE |
| 440440925260000 | ST TREATMENT SWALLOW |
| 440440925260001 | ST TREATMENT SWALLOW 15 MIN |
| 440440925970000 | ST EVAL ORAL SPEECH DEVICE |
| 440440925990000 | ST PROCEDURE UNLISTED |
| 440440926260000 | ST EVAL AUDITORY REHAB STATUS 1ST HOUR |
| 440440926270000 | ST EVAL AUDITORY REHAB STATUS EA ADDL 15 MIN |
| 440440926300000 | ST AUDITORY REHAB PRE-LINGUAL HEARING LOSS |
| 440440926330000 | ST AUDITORY REHAB POST-LINGUAL HEARING LOSS |
| 440440926400000 | ST ANALYSIS PROGRAM AUDITORY BRAINSTEM IMPL PER HR |
| 440440927000000 | ST UNLISTED PROCEDURE |
| 440440927000001 | ST UNLISTED PROCEDURE 15 MIN |
| 440440975320001 | ST COGNITIVE TRAINING 15 MIN |
| 440440975330001 | ST SENSORY PERCEPTION 15 MIN |
| 440441926060000 | ST TREATMENT NON-SPEECH-GENERATING DEV USE |
| 440441926090000 | ST TREATMENT SPEECH-GENERATING DEV USE |
| 440443925080000 | ST TREATMENT SPEECH GROUP |
| 440443925080001 | ST TREATMENT LANGUAGE GROUP 15 MIN |
| 440443925080002 | ST TREATMENT SPEECH GROUP 15 MIN |
| 440443925080003 | ST TREATMENT SPEECH GROUP PED 15 MIN |
| 440444000500000 | ST CONSULT 15 MIN |
| 440444925060000 | ST EVAL |
| 440444925060001 | ST EVAL SPEECH PED 90 MIN |
| 440444925060002 | ST RE-EVAL SPEECH 15 MIN |
| 440444925060003 | ST EVAL SPEECH 15 MIN |
| 440444925060004 | ST EVAL SPEECH PED 15 MIN |
| 440444925060005 | ST EVAL SCREENING 15 MIN |
| 440444925060006 | ST EVAL AUDITORY |
| 440444925060007 | ST EVAL SPEECH 60 MIN |
| 440444925060008 | ST EVAL VOICE |
| 440444925060009 | ST EVAL SPEECH PED |
| 440444925060010 | ST EVAL SPEECH 30 MIN |
| 440444925060011 | ST RE-EVAL SPEECH 30 MIN |
| 440444925250000 | ST EVAL SWALLOW 15 MIN |
| 440444925250001 | ST EVAL SWALLOW VIDEO/FLUORO |
| 440444925250002 | ST EVAL SWALLOW 60 MIN |
| 440444925250003 | ST EVAL ORAL FUNCTION |
| 440444925250004 | ST EVAL SWALLOW VIDEO/FLUORO 15 MIN |
| 440444925250005 | ST EVAL SWALLOW 30 MIN |
| 440444926050000 | ST EVAL FOR NON-SPEECH-GENERATING DEVICE |
| 440444926070000 | ST EVAL FOR SPEECH-GENERATING DEVICE 1ST HR |
| 440444926080000 | ST EVAL FOR SPEECH-GENERATING DEV EA ADD 30MIN |
| 440444926100000 | ST EVAL SWALLOW |
| 440444926110000 | ST EVAL SWALLOW FLUORO CINE/VIDEO |
| 440444926120000 | ST EVAL SWALLOW ENDO CINE/VIDEO |
| 440444926140000 | ST EVAL ENDO SENSORY TEST CINE/VIDEO |
| 440444926160000 | ST EVAL ENDO SWALLOW/SENSORY TEST CINE/VIDEO |
| 440444926180000 | ST EVAL FOR NON-SPEECH-GENERATING DEVICE ADDL |
| 440444926200000 | ST EVAL CENTRAL AUDITORY FUNCTION 1ST HR |
| 440444926210000 | ST EVAL CENTRAL AUDITORY FUNCTION EA ADDL 15 MIN |
| 440444926250000 | ST ASSESSMENT OF TINNITUS |
| 440444961050000 | ST ASSESSMENT APHASIA PER HR |
