# Supplementary figures and images for: Patterns of utilization and effects of hospital-specific factors on physical, occupational, and speech therapy for critically ill patients with acute respiratory failure in the USA: results of a 5-year sample
Source: Crit Care. 2019 May 16;23:175. doi: 10.1186/s13054-019-2467-9 (PMC6524324; doi:10.1186/s13054-019-2467-9)

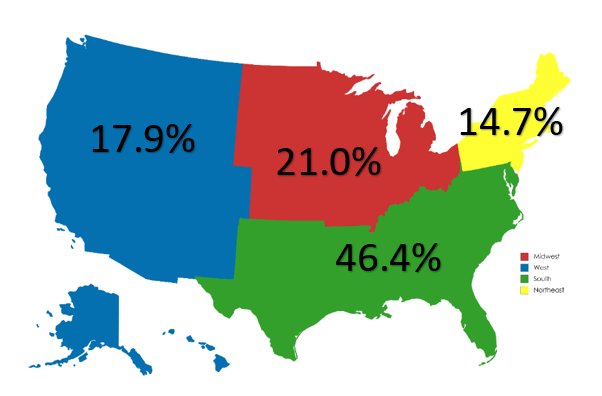

Supplement: Supplementary file 3 — Figure S1. Geographic regions. (DOCX 65 kb) [file 13054_2019_2467_MOESM3_ESM.docx]
